# Do False Memories Look Real? Evidence That People Struggle to Identify Rich False Memories of Committing Crime and Other Emotional Events

**DOI:** 10.3389/fpsyg.2020.00650

**Published:** 2020-04-08

**Authors:** Julia Shaw

**Affiliations:** Psychology and Language Sciences, University College London, London, United Kingdom

**Keywords:** false memory, legal psychology, memory, confessions and false confessions, forensic psychology and legal issues

## Abstract

Two studies examined whether people could identify rich false memories. Each participant in both studies was presented with two videos, one of a person recalling a true emotional memory, and one of the same person recalling a false memory. These videos were filmed during a study which involved implanting rich false memories (Shaw and Porter, 2015). The false memories in the videos either involved committing a crime (assault, or assault with a weapon) or other highly emotional events (animal attack, or losing a large sum of money) during adolescence. In study 1, participants (*n* = 124) were no better than chance at accurately classifying false memories (61.29% accurate), or false memories of committing crime (53.33% accurate). In study 2, participants (*n* = 82) were randomly assigned to one of three conditions, where they only had access to the (i) audio account of the memory with no video, (ii) video account with no audio, or (iii) the full audio-visual accounts. False memories were classified correctly by 32.14% of the audio-only group, 45.45% of the video-only group, and 53.13% of the audio-visual group. This research provides evidence that naïve judges are not able to reliably identify false memories of emotional or criminal events, or differentiate true from false memories. These findings are likely to be of particular interest to those working in legal and criminal justice settings.

Can people tell whether a particular memory is true or false? In a review of the literature, researchers have pointed out that there are two ways of looking at this question – “focusing on the memories reported or the person reporting the memories” (Bernstein and Loftus, 2009, p. 370). Within this review, it was argued that there were no reliable neurophysiological, technological, or psychological ways to discern between true and false memories – and that telling the difference between true and false memories is one of the biggest challenges in memory research. However, this hasn’t stopped researchers from continuing to look for differences, with limited success.

Some researchers have argued that the phenomenology of false memories is different from true memories, advocating that participants are able to identify their own false memories if they focus on source monitoring decisions (where people think they know things from), confidence ratings, and explicit warnings about memory fallibility (Anastasi et al., 2000). Others have argued that providing questionnaires that help people systematically examine the characteristics of their memories can slightly improve false memory detection (Ost et al., 2002). Proponents of this phenomenological line of work broadly argue that true memories feel “richer” than false ones (Marche et al., 2010), and that false memories are “weaker” forms of true memories (Jou and Flores, 2013).

However, this seems an incomplete answer to the differences between true and false memories, as research also shows that the realism of false memories depends on the method through which they were generated (Jou and Flores, 2013). Most studies on false memories involve short timeframes, and false memories that are neither very complex, nor particularly emotional. Research has also focused almost entirely on assessments of one’s own false memory account, rather than assessments of someone else’s account. Research shows that the methodologies that use longer encoding periods, repetition, emotion, and a lot of detail and complexity create false memories that feel and look more real (Jou and Flores, 2013). Such methodology is typical of studies that try to implant rich false memories of autobiographical events, through a method called the familial informant false narrative paradigm (Loftus and Pickrell, 1995). This technique involves using a combination of trust, misinformation, imagination exercises, and repetition to convince participants that they experienced events that never happened. By using this technique, individuals have been shown to generate complex false memories of autobiographical events (Scoboria et al., 2017).

An autobiographical false memory is an incorrect recollection of part of an event, or an incorrect recollection of an entire event. The person recalling a false memory believes that they are accessing a real memory – it is not an attempt to lie (e.g., Loftus, 2005). Memories that have been implanted using the familial informant false narrative technique – and related techniques – include getting lost in a shopping mall (Loftus and Pickrell, 1995), spilling a punch bowl at a family wedding or being left in the car as a child and releasing the parking break so it rolled into something (Hyman et al., 1995). More serious false memories that have been implanted include being punched or punching someone else (Laney and Takarangi, 2013), or being the victim of an animal attack (Porter et al., 1999). Additionally, researchers have implanted a number of false memories of committing crime, including of assault, assault with a weapon, and theft (Shaw and Porter, 2015). Rich false memories of highly emotional or criminal events are of particular interest to applied psychologists, legal professionals, and law enforcement, as they can have catastrophic consequences. Because they can become distorted or fabricated evidence, such false memories can seriously threaten the integrity of a criminal investigation or legal case (e.g., Loftus, 2003).

Research on autobiographical false memories typically involves asking the participants themselves to rate the realism of their own (false) memories, and participants consistently report that such false memories feel incredibly real (e.g., Shaw and Porter, 2015; Scoboria et al., 2017). If autobiographical false memories feel largely the same as real memories, then they may also look like real memories to others. In perhaps the only study to directly examine this, participants were asked to watch videos of complex emotional true and false memories being recalled, to see if they could tell the difference (Campbell and Porter, 2002). Observers correctly identified 60% of false memories, and 53% of true memories – with 50% representing chance. This study was the inspiration for the present research. While there has been evidence to show that false memories of important emotional and criminal events can be created (e.g., Shaw and Porter, 2015; Scoboria et al., 2017), there has been little research investigating the ability of observers to distinguish between true and false memories, and no evidence on false memories of crime.

Two studies examined whether participants could correctly identify false memories. The three main hypotheses were (H1) people are no better than chance at identifying false memories, (H2) people are no better than chance at identifying false memories of criminal events, (H3) people are better at comparative judgments than absolute ones (once they know one of two memories is false, they can identify the “richer” memory). Study 2 adds an exploratory component to this, to examine whether it would make a difference if people could only see (video with no audio), hear (audio with no video), or see and hear (video with audio) the false memory accounts. This was examined for two reasons. First, it is possible that visual cues are distracting, so participants might be better able to identify false memories when they only have audio and can focus on content. Conversely, in Campbell and Porter (2002) memory classification accuracy was better for those who relied on non-verbal cues, so perhaps verbal or content cues are distracting, which could make it easier to identify false memories without sound. Additionally, evidence in legal cases is sometimes only available as audio recordings or as video footage with no sound, so examining this issue likely has practical applications. The present studies further our understanding of the realism of false memories, and whether false memories can be identified by observers.

## Materials and Methods

### Participants

#### Study 1

Participants were recruited for a study called “evaluating emotional memories” and told “The purpose of this project is to examine whether participants are able to distinguish between different kinds of memories.” Participants were recruited through posters that indicated entry into a $50 draw, and from the University of British Columbia Okanagan (Canada) research pool. Participants (*n* = 124) completed the study between January and March 2013. Most identified as women (*n* = 103), 21 as men. Age categories were provided, and 116 participants were age 18 to 24, the rest were over 25. The categories from the Canadian Census at the time were adopted; of the participants 88 were White, 14 Chinese, 7 South Asian, 7 Southeast Asian, 2 Aboriginal, 2 Black, 2 Filipino, 1 Japanese, and 1 Korean. Almost all were undergraduate students (*n* = 122). Mean number of psychology courses taken was 3.694 (*SD* = 3.121). Participants were asked whether they had taken any related classes – 104 indicated they had never taken a course on memory, 110 had never taken a forensic psychology course, 97 had never taken either.

#### Study 2

Participants were recruited to “participate in memory research” through emails and posters on the University of Bedfordshire campus (United Kingdom), where they could enter a draw to win one of four £50 prizes. Participants (*n* = 82) completed this study between February 2014 and May 2015, of these 61 identified as women, 21 as men, and one as neither. The mean age was 22.13 years (Range = 18–43, *SD* = 5.871). The breakdown from the United Kingdom government at the time was used to measure ethnicity; 46 participants were White, 16 Asian, 16 Black, 3 Mixed-race, and 1 person did not specify. Most participants were undergraduate students (*n* = 77), 4 were masters students, and 1 had a PhD. Most participants (*n* = 56) had previously taken a course on memory, 18 on forensic psychology, and 21 had taken neither.

### Design

#### Study 1

Participants were randomly assigned to one of two conditions; to watch a video of false memory of an emotional event or a crime. The influence of the independent variable ‘type of memory’ on the dependent variable ‘classification accuracy’ was measured.

#### Study 2

Participants were randomly assigned to one of three conditions; to watch memory videos with audio and video, as audio-only (with no video), or video-only (no audio). The influence of the independent variable ‘media type’ on the dependent variable ‘classification accuracy’ was measured.

### Materials

#### Study 1 and Study 2

This research used videos collected by Shaw and Porter (2015). The eight participants whose videos were used provided permission for the interviews to be used in future research. The videos used for the present research involve each participant recalling two separate accounts in structured interviews. One of the emotional autobiographical events described actually occurred during the participants’ adolescence (between the ages of 11 and 14), information about which was obtained from the participants’ parents. The second account was generated through the familial informant false narrative procedure (Loftus and Pickrell, 1995), and each account was classified as a rich false memory by Shaw and Porter (2015). The false memories involved accounts of emotional or criminal events from the participants’ adolescence, the events allegedly happened between the ages of 11 and 14, and those recalling them were on average 20 years old. All videos included were also classified as false memories in the Shaw and Porter data re-analysis by Wade et al. (2018). For a discussion of this coding disagreement see Shaw (2018).

Four criteria were used to select these eight participants from the 60 who took part in the original Shaw and Porter (2015) study. (1) The participants recalled a diverse set of false memories, including complex emotional and criminal events occurring during adolescence. (2) The true and false memories told by the participant were of a similar length, as to minimize length as a confound. (3) Half of participants were selected to be female and half male, to account for potential gender effects. (4) The nature of the false memories for the men and women was selected to be comparable.

Each participant in the present studies saw the same person recalling a true and a false memory. This was done because there are individual differences in how individuals recall accounts. Had videos from different individuals been used (e.g., showing a man in one video and a woman in the other), it is likely that the participants would have been distracted by differences between *who* was recalling the account, rather than focusing on *what* they were recalling and whether the accounts were true or false. See Table 1 for a brief description of the nature of each video set used.

**TABLE 1:** Content of false memory and true memory videos used for both studies.

Set	Gender	False memory	True memory
**1**	Female	Assault with police contact	Physical injury, had to get stitches
**2**	Male	Assault with police contact	Stranded at closed school in winter
**3**	Female	Assault with a weapon, with police contact	Physical injury, had to get stitches
**4**	Male	Assault with a weapon, with police contact	Day mother passed away from cancer
**5**	Female	Attacked by a vicious animal	Frightening tooth surgery
**6**	Male	Attacked by a vicious animal	Day the family dog passed away
**7**	Female	Lost a large sum of money and parents were upset	Distressed when told moving to a new home
**8**	Male	Lost a large sum of money and parents were upset	Bullied and threatened by a peer online

*Participants in both study 1 and study 2 were randomly assigned to watch one of eight sets of videos, each involving the same person recalling a true and a false emotional memory. The gender of the person recalling the memories in each set is noted here, as is the content of their true memory and their false memory. For study 1, the “criminal” condition involved videos 1–4 and the “emotional” condition involved videos 5–8. For study 2, participants in all conditions were randomly assigned to any of the eight sets.*

### Procedure

#### Study 1

Ethical clearance was granted by the University of British Columbia Okanagan research ethics board (reference: H12-03340). Participants scheduled an appointment using the university participant recruitment tool to participate in a lab-based study. This system enabled automatic exclusion of participants who had been part of the related, previous, false memory study that was conducted on the same campus (Shaw and Porter, 2015). Once in the lab, participants were given a consent form, and all study procedures were explained to them.

Next, participants were randomly assigned to one of two conditions – to watch a false memory that was criminal or one that was emotional. In each condition, participants watched a video of an individual recalling a false memory and video of the same individual recalling a true memory. Videos were counterbalanced.

Immediately before each video was viewed, all participants were told;

“All, some, or none of the videos you are about to watch involve memories of real accounts. Your task is to identify after each video whether you think the account described actually happened or not. Consider each video carefully, and make note of any cues you are using to make your decision. These cues can involve the content of the accounts provided, verbal or behavioral cues, or any other cues you think are relevant.”

This instruction was crafted with generalisability in mind. It is rare that individuals are asked outside of a research setting whether an account is a false memory, but it is common to ask whether someone thinks a described event really happened. It is possible that some participants took this instruction to mean that they should evaluate whether the individual is lying – if so then the same individuals would likely also do this when faced with a similar task outside the lab. This is also true for legal settings. If an eyewitness or defendant describes an event, the key question by police or lawyers is usually “did this happen” rather than “is this a false memory.”

Participants in study 1 were asked at the beginning of the first video if they had seen the individuals depicted in the videos before, as those in the videos were students on the same campus. If they said yes, participants were randomly given a new set of videos (this was not recorded by the research assistants, but anecdotally it was only necessary once).

Participants spent about 10 min watching video 1, then were asked questions about video 1, then spent about 10 min watching video 2, and were asked questions about video 2. After each video, participants were asked to give an absolute judgment regarding whether the video they just watched actually happened. Participants were then asked to select all that applied from a list of cues that they may have used to make their decision, synthesized from cues often cited as being related to identifying false memories and deception. Although it might be clear looking at the list of items that these were based partly on the deception detection literature, this was unlikely to be noticed by lay participants as the items were broad. Participants were also asked to rate how confident they were in each decision, by selecting an integer between 0 (not confident) and 100 (entirely confident).

After viewing both of the videos participants read “One of the videos you watched involved a real memory and one of the videos involved a false memory.” And they were asked which one they thought was false. The participants were not able to review the videos a second time to aid in their decision, and the participants were asked to identify the cues that they used to make this comparative decision, and their confidence in it. Although this is not an ecologically valid situation, as individuals almost never have enough ground truth for memories to know that one of two memories is false, this was done to see whether the ability to compare two memories would make it easier to identify a false memory. Finally, participants were asked to complete a demographics questionnaire and debriefed.

#### Study 2

The method for study 2 was almost identical to study 1. The only methodological modification was the conditions to which participants could be assigned. Participants were randomly assigned to one of three conditions. In condition one (audio-visual), participants watched a video with sound randomly selected from one of the eight sets shown in Table 1. This condition served as a replication of study 1. In condition two (audio only), participants were asked to listen to one of the eight sets of videos, but they could only hear the audio recordings from the videos with no picture. In condition three (video only), participants were asked to watch one of the eight sets of videos, but could only see the recording of the videos with no sound.

Ethical clearance for study 2 was granted by the University of Bedfordshire research ethics board (reference: “Differentiating between true and false emotional memories”). Two research assistants ran all participants in a lab space on the University of Bedfordshire campus.

## Results

Data were analyzed using the open source software JASP Team (2019). All Bayes Factors were interpreted as described by Jarosz and Wiley (2014) (and originally suggested by Raftery, 1995) using the recommended labels: weak (inverse Bayes factor: 1–3), positive (3–20), strong (20–150), or very strong evidence (>150). All data are available in Supplementary Material.

### False Memories

Participants classified 57.26% of false memory accounts correctly in study 1, and 43.90% in study 2. A Bayesian multinomial test with expected proportions was conducted for each study separately. Evidence for both was in favor of the null hypothesis that participants score no different from chance when classifying false memories: study 1 provided weak support for this (BF_01_ = 2.44), and study 2 provided positive support for this (BF_01_ = 3.99). Table 2 displays the percentage of participants for each condition for both studies who classified the memory videos correctly and incorrectly.

**TABLE 2:** Participant classification accuracy for true memories and false memories.

	True memory		False memory	

Condition	Correct	Incorrect	Correct	Incorrect
**Study 1**				
Emotional (*n* = 60)	68.75%	31.25%	59.38%	40.63%
Criminal (*n* = 64)	53.33%	46.67%	55.00%	45.00%
Overall (*n* = 124)	61.29%	38.71%	57.26%	42.74%
**Study 2**				
Audio-Visual (*n* = 32)	62.50%	37.50%	53.13%	46.88%
Video only (*n* = 22)	68.18%	31.82%	45.45%	54.55%
Audio only (*n* = 28)	60.71%	39.29%	32.14%	67.86%
Overall (*n* = 82)	63.41%	36.59%	43.90%	56.10%

*Borrowing the language commonly used for signal detection theory, this table shows the hits (true memories correctly classified as true), misses (true memories incorrectly classified as false), correct rejections (false memories correctly classified as false), and false positives (false memories incorrectly classified as true) broken down by the condition participants were assigned to.*

### False Memories of Crime

Criminal false memories were classified correctly by 55.00% of participants in study 1, and 43.59% in study 2. A Bayesian multinomial test with expected proportions was conducted for each study separately. Evidence from both studies was in favor of the null hypothesis that participants scored no better than chance at accurately classifying false memories of crime: study 1 provided weak support for this (BF_01_ = 2.12), and study 2 provided positive support for this (BF_01_ = 3.71).

### Media Type

In study 2 the type of media participants engaged with varied. Participants were most accurate when they saw videos with audio, with 53.13% correctly classifying false memories, and worst (32.14%) when they were given only audio. A binomial logistic regression was conducted and no significant association between media type and accuracy for absolute memory judgments was found (χ2 = 1.08, *p* = 0.298, specificity = 73.9%, sensitivity = 27.8%).

### Comparative Judgments

After both videos were rated (absolute judgments), participants were asked to judge which one of the two was false (comparative judgment). Two Bayesian multinomial tests with expected proportions were conducted, separately for study 1 and study 2. Evidence for both studies was strongly in favor of the hypothesis that participants were classifying memories different from chance. However, the direction of the difference was opposite. In study 1, 64.52% of participants correctly comparatively identified the false memory (BF_10_ = 21.49), while in study 2 only 31.71% did so (BF_10_ = 35.14).

### Confidence

Confidence for both studies was rated by participants as an integer between 0 and 100, and accuracy was binary (correct/incorrect). Two binomial logistic regressions were completed, one for each study. Results indicate a significant association between confidence and accuracy across all decisions (combining decisions for video 1, video 2, and the comparative judgment) for study 1 (χ^2^ = 9.58, *p* = 0.002, specificity = 15.8%, sensitivity = 92.9%), but not for study 2 (χ^2^ = 3.76, *p* = 0.056, specificity = 69.7%, sensitivity = 41.2%).

### Cues

**FIGURE 1:**
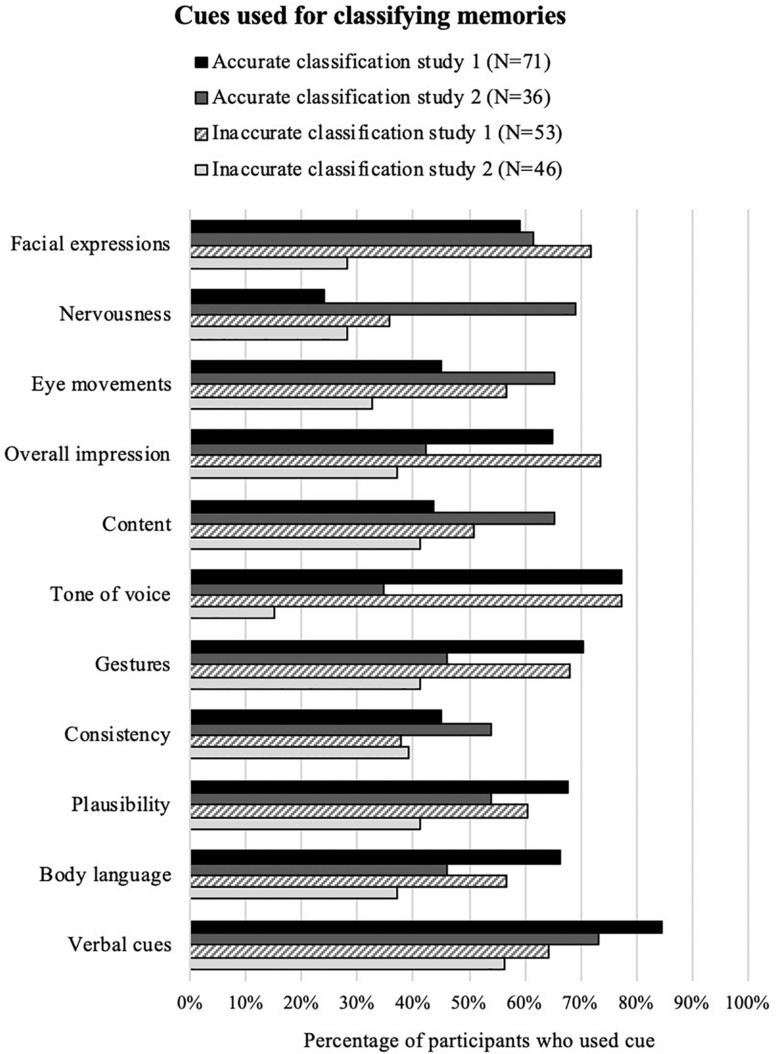
Cues participants indicated they used when deciding how to classify false memories for both studies. This table includes percentages of the people who chose each cue taking into account (omitting) those who had no access to those cues. For example, omitting audio-only participants from the calculation for the percentage of “tone of voice” cue.

Participants could indicate whether they relied on specific cues when judging the memories. Figure 1 shows the breakdown of cues participants indicated they relied on when classifying false memories for study 1 and study 2, broken down by whether the participants accurately classified them as false, or inaccurately classified them as true. The largest difference for study 1 was for verbal cues, so a chi-squared test was conducted to examine this relationship and a significant but weak positive relationship was found, *x*^2^(1, *n* = 124) = 6.86, *p* = 0.009, Φ = 0.235, but the same relationship was not found for study 2, *x*^2^(1, *n* = 82) = 0.114, *p* = 0.735, Φ = 0.037. No cues were consistently related to accuracy.

### Order Effects

A Bayesian binomial test with expected proportions tested against 0.5 was conducted for each study. For study, 1 evidence was in favor of the null hypothesis that participants did not have a response bias for either video 1 (weak support, BF_01_ = 1.28) or video 2 (positive support, BF_01_ = 4.07). The same was true for study 2, where both video 1 and 2 had the exact same result (positive support, BF_01_ = 5.87).

## Discussion

This research provides evidence that false memories look real. Participants were no better than chance at identifying rich emotional false memories, and no better than chance at identifying rich false memories of committing crime. These results are in line with previous research (Campbell and Porter, 2002), and support the sentiment that false memories feel real, so they should look real (Bernstein and Loftus, 2009). The present research adds to the literature in two ways; it was the first to investigate observer accuracy for (i) rich false memories of crime, and (ii) rich false memories of adolescent events (most rich false memories implanted by researchers are of early childhood events).

Even when participants knew that one of the memories was false and the other true, they were unable to reliably tell the difference. While in study 1 comparative judgments were more accurate than chance, in study 2 they were less accurate than chance. This suggests that perhaps there is no real difference between absolute and comparative judgments, but future research is needed to clarify this.

Whether participants were hearing, seeing, or hearing and seeing the accounts, participants did not score significantly better or worse than chance. However, the pattern of results suggests that it is possible that audio-only false memory accounts would be found to be at highest risk of being misclassified as true memories. This would benefit from further examination with a larger sample as there are some situations, particularly in legal contexts, where an audio recording may be the only available evidence. If people are significantly more likely to judge false memories as true in such contexts this could present a risk.

Did participants who correctly classified memories as false rely on any particular cues to do so? Previous research on false memory classification showed that accurate judges reported using more cues overall, and more non-verbal cues, than inaccurate judges (Campbell and Porter, 2002). This was not found in the present research. In all conditions, across both studies, self-reported cues used to make the memory judgments showed no informative patterns. This may help to explain the finding that participants were no better than chance at identifying false memories – because the cues they relied on were either uninformative, counter-productive, or both. This is consistent with related research on deception detection, which shows that individuals often rely on misconceptions and ineffective cues when deciding whether an account is true or false (Levine, 2018).

A possible limitation is that the studies were conducted between 2013 and 2015, so replication using a contemporaneous sample would be helpful as awareness about the existence of false memories may have further permeated social consciousness. That being said, there is no evidence that participants who know more about false memories can better identify a memory as false simply by looking at an account, and there is some evidence that exposure to educational material on false memories can impair judgment (Campbell and Porter, 2002). Future studies could also consider looking at the ability to identify false memories with more diverse, and non-student, populations.

The results presented here have direct implications for police and legal contexts. In addition to the risk of misidentifying false memories as true, the results presented here show the risk of misidentifying true memories as false – participants were no better than chance at correctly classifying true memories. In legal contexts, if the issue is raised that a particular witness is mistaken, this suggests that people may quite readily accept that a true memory is false, or that a false memory is true. Integrating this insight into legal documents, expert reports, and training for police and other investigators would be useful. Such understanding would help individuals be skeptical about their own assumption as to whether a particular memory is true or false, and help to contextualize memory evidence when presented to judges, juries, and investigators. Overall, this research is in line with findings by Bernstein and Loftus (2009), and the answer to the question: “Do false memories look real?” continues to be “yes.”

## Data Availability Statement

All datasets generated for this study are included in the article/Supplementary Material.

## Ethics Statement

The studies involving human participants were reviewed and approved by Study 1: University of British Columbia Okanagan research ethics board (reference: H12-03340), and Study 2: University of Bedfordshire research ethics board (reference: “Differentiating between true and false emotional memories”). The participants provided their written informed consent to participate in this study.

## Author Contributions

The author confirms being the sole contributor of this work and has approved it for publication.

## Conflict of Interest

The author declares that the research was conducted in the absence of any commercial or financial relationships that could be construed as a potential conflict of interest.
